# Developmental Perchlorate Exposure: Gilbert Responds

**DOI:** 10.1289/ehp.0800532R

**Published:** 2009-06

**Authors:** Mary E. Gilbert

**Affiliations:** Neurotoxicology Division U.S. Environmental Protection Agency Research Triangle Park, North Carolina E-mail: gilbert.mary@epa.gov

I welcome the opportunity to respond to the comments of Mavis and DeSesso regarding our article on the developmental effects of perchlorate exposure on brain function (Gilbert and Sui 2008). Although couched in strong terms, their criticisms are not pertinent to the underlying physiologic processes that we investigated, and many of the points they raised were addressed in our original article. The comments of Mavis and De Sesso alter neither the significance of our study nor the integrity of our conclusions.

Mavis and DeSesso opine that the “evidence for ‘thyroid hormone insufficiency’ is questionable.” They base this opinion on two points. First, they argue that there is no “dose–response” effect of perchlorate, and second, they focus on serum triiodothyronine (T_3_) as the biologically active hormone. They completely ignore the effect of perchlorate in the dams, and they do not acknowledge that circulating levels of T_3_ do not reflect thyroid function. More than 80% of serum T_3_ is derived from peripheral deiodination of thyroxine (T_4_) and is not tightly linked to thyroid hormone action in the developing brain. Tissue (not serum) concentration of T_3_, as well as the critical window over which the T_3_ is required, dictates the nuclear action of thyroid hormone. Different tissues, including brain, can be deficient in T_3_ while serum levels of this hormone are unchanged. In addition T_4_ and other iodo thyronines interact with membrane-bound hormone receptors and can directly affect, through non nuclear actions, the biological activity in the cells of many tissues. Mavis and DeSesso also inaccurately limit the discussion of thyroid hormones and tissue function to circulating serum levels of T_3_ in developing pups.

Mavis and DeSesso confuse analytical variability of the hormone measures with biological effect size: One characterizes the precision and accuracy of the specific assay (inter- and intra-assay variation); the other characterizes the variation among animals attributable to treatment. The performance of the T_3_ and T_4_ assays we reported (Gilbert and Sui 2008) fall well within the manufacturer’s recommended limits, and variation between replicate samples was typically < 3%. Although thyroid-stimulating hormone (TSH) assays are inherently more variable, performance also fell within acceptable limits (9–12%). These sources of variance, as Mavis and DeSesso portend, do not under lie the reported effects on serum hormones in pups.

The consequences of small reductions in serum T_4_ on brain development in pups and dams are not trivial and should not be dismissed (for review, see Zoeller and Rovet 2004). Unless perchlorate is acting through a non thyroidal mechanism, our study fully supports recent data indicating that small changes in maternal and/or neonatal serum thyroid hormone can impact brain development.

We are perplexed by Mavis and DeSesso’s erroneous characterization of *in vivo* field potentials as “electrophysiologic anomalies.” This seems to reflect a misconception of the value of these measures as functional indices of integrated physiologic responses in an intact neural circuit. These end points are widely acknowledged to reflect the functional integrity of the hippocampus. Our data (Gilbert and Sui 2008) go beyond the more commonly reported acute response in an isolated hippocampal brain slice to reveal impairments in the fundamentals of neuronal communication in living animals, and the changes were demonstrated months after exposure to perchlorate had ceased. Certainly a permanent impairment in the synaptic function of any brain structure must be considered an adverse neurotoxicologic insult. Input/output (I/O) functions [Figure 4 (Gilbert and Sui 2008)] reflect the ability of a population of neurons to transmit signals across a monosynaptic connection. Synaptic plasticity, the ability to adapt to stimuli, tested in the form of paired pulse (PP) depression and facilitation measure the influence of local circuit neurons to modulate that synaptic output [Figure 5 (Gilbert and Sui 2008)]. The clear relationship between increasing stimulus strength and increases in the amplitude of the physiologic response, evident in both the I/O and PP data, validates the high degree of experimental control maintained over these biological responses. Contrary to the allegations of Mavis and DeSesso, both sets of meas ures demonstrate dose-dependent perturbations as a function of perinatal perchlorate exposure and represent important contributions to a literature largely lacking examinations of dose–response relationships. Furthermore, these findings are in complete agreement with observations using graded levels of the known goitrogen propylthiouracil (PTU) over a similar dosing regimen.

Mavis and DeSesso state that electrophysiologic anomalies are not evidence of neurologic impairment as they occurred in the absence of behavioral changes (Gilbert and Sui 2008). As discussed in our article, the neuroscience literature holds many instances where molecular, neurochemical, anatomical, and electro physiologic indices do not correlate with apical behavioral measures. This does not negate the significance of these downstream observations, but rather reveals the relative bluntness of some of the behavioral tools available to assess cognitive function in rodent models. Attempts to evaluate subtle perturbations of the thyroid axis will require further refinement of existing paradigms to increase sensitivity or the utilization of more sophisticated evaluations of behavioral dysfunction. This paradigm shift is not dissimilar from what was necessary in the behavioral evaluation of developmental lead exposure two decades ago.

Mavis and DeSesso state that the concentrations used in our study (Gilbert and Sui 2008) bear no relevance to the human health risk assessment for perchlorate. However, the purpose of our study was not to emulate human exposures to perchlorate. Rather, percholorate was used to disrupt the thyroid axis via a mechanism distinct from standard model compounds (i.e., PTU and methimazole) to examine the impact of mild perturbations of the thyroid axis on neurodevelopment. Nonetheless, the results revealed a significant reduction in synaptic function at a dose (30 ppm = 4.5 mg/kg body weight per day) consistent with the lowest observable adverse effect levels identified from the review of all available animal data and summarized in the U.S. Environmental Protection Agency’s perchlorate risk assessment document (U.S. EPA 2002). As such, these findings corroborate previous findings and add additional weight to the existing evidence from animal studies on the negative impact of perchlorate on brain development.

Finally, according to Mavis and DeSesso, the rat model is questionable for sensitivity of the human thyroid system to perchlorate because of differences in thyroid hormone storage. The hypothalamic–pituitary–thyroid axis is very similar in its chemistry and its function in rodents and humans, and rodent models have provided important information on the fundamental biology of endocrine systems informing medical practice and public health protection. Although differences in thyroid hormone economy of adult rats make rodents less than ideal for the assessment of thyroid tumors, the dependence of the fetus on the maternal supply for thyroid hormone makes the rat a suitable model for neurodevelopment. In humans, differences in the capacities and the relative immaturity of compensatory mechanisms of the fetus and neonate increase the vulnerability of these life stages and have significant implications for tolerance to perturbations of the thyroid axis. Rather than detracting from the utility of the model, the limited storage capacities of the rodent offer a reasonable parallel to the immature human.

In conclusion, the comments of Mavis and DeSesso are not consistent with current thinking in the fields of thyroid endocrinology or neuroscience. Their critique does not effectively challenge the veracity of our observations or the soundness of our conclusions.

## References

[b1-ehp-117-a237] Gilbert ME, Sui L (2008). Developmental exposure to perchlorate alters synaptic transmission in hippo campus of the adult rat. Environ Health Perspect.

[b2-ehp-117-a237] U.S. EPA (2002). Perchlorate Environmental Contamination: Toxicological Review and Risk Characterization (2002 External Review Draft) NCEA-1-0503.

[b3-ehp-117-a237] Zoeller RT, Rovet J (2004). Timing of thyroid hormone action in the developing brain: clinical observations and experimental findings. J Neuroendocrinol.

